# Sub-THz Characterization of Technical Surfaces for Particle Accelerator Vacuum Chambers

**DOI:** 10.3390/s24155036

**Published:** 2024-08-03

**Authors:** Andrea Passarelli, Maria Rosaria Masullo, Zahra Mazaheri, Antonello Andreone

**Affiliations:** 1 National Institute for Nuclear Physics-Naples Unit, Monte Sant’Angelo University Complex, Via Cintia, 80126 Naples, Italy; masullo@na.infn.it (M.R.M.); zahra.mazaheri@unina.it (Z.M.); andreone@unina.it (A.A.); 2 Department of Physics “E. Pancini”, University of Naples “Federico II”, Monte Sant’Angelo University Complex, Via Cintia, 80126 Naples, Italy

**Keywords:** THz, waveguide spectroscopy, coating materials, particle accelerators

## Abstract

Coatings play a crucial role in the functionality of vacuum chambers in particle accelerators, serving a dual goal by efficiently facilitating pumping and mitigating electron cloud effects. However, their impact on the surface impedance of the chamber walls raises concerns, potentially affecting the machine performance and imposing limitations on achievable energies and currents. Therefore, an electromagnetic characterization is essential for a comprehensive study of accelerator structures, particularly in the context of the next-generation machines where the demand for extremely short particle bunches accentuates the importance of evaluating material responses in the very-high-frequency region. We present a technique for probing the sub-THz response of coating materials by measuring pulsed signals passing through a specifically designed waveguide, in which is placed a slab with the deposited material under test. The proposed methodology allows for a comprehensive exploration of the electromagnetic properties of the most used technical surfaces (substrate plus coatings) in accelerators under realistic conditions, providing valuable insights into their behavior in the sub-THz frequency range. The experimental data of three different Non-Evaporable Getter coating samples, prepared on a copper substrate at the CERN deposition facilities under different sputtering conditions, are discussed. The findings contribute to a deeper understanding of the complex interactions between coatings and accelerator structures, with the aim of optimizing performance and efficiency in the evolving landscape of particle acceleration technologies. The limitations and advantages of the technique are also reported.

## 1. Introduction

Collective effects, which arise from the electromagnetic (EM) interactions of beam particles with themselves and the surrounding vacuum chamber, change the particle distribution. These interactions may lead to beam instabilities and can significantly limit the performance of new-generation particle accelerators, colliders as well as light sources in terms of beam intensity, luminosity, and brightness. Therefore, mitigation techniques to improve these limitations are also evaluated.

In this framework, novel accelerators will require special treatment of the vacuum chamber (pipe) surface to prevent electron cloud (e-cloud) effects. These effects are caused by the generation of secondary electrons from the pipe wall due to hitting electrons created by the ionization of residual gas or photo-electrons from synchrotron radiation [1]. In recent years, extensive research has been conducted to identify the best possible candidates for internal pipe surface coatings. Non-Evaporable Getter (NEG) alloys have emerged as a promising solution [2]. These alloys can be deposited on the inner walls of vacuum pipes, transforming the chamber into an efficient pump. NEG films also reduce induced gas desorption and secondary electron yields. However, applying any coating material to an accelerator, whether for e-cloud reduction, vacuum improvement, or both, inevitably alters the overall surface impedance. This change can potentially cause beam instability due to electromagnetic interactions with the surroundings [3,4,5]. Therefore, it is crucial to perform accurate EM characterization of the coating material before its application in the beam pipe. This ensures the development of a reliable impedance model and helps identify potential issues and performance limitations in modern particle accelerators and storage rings [6].

Various alternative approaches have been used for the characterization of thin films or coatings, which depend on the type of information, related to physical, electronic, and chemical properties, one wishes to retrieve. Moreover, in the case of particle accelerators, the characterization frequency bandwidth is strongly related to the particle bunch length. The influence of coatings will be larger at higher frequencies in the case of shorter bunches [7]. A classical method for the high-frequency characterization of thin films, described in [8], involves the use of a RF test cavity. In this work, the bulk conductivity of two types of NEG films (dense and columnar) is achieved by measuring their surface resistance retrieved by the cavity resonance at 7.8 GHz. In [9], the study focused on the resonance properties of NEG coated and uncoated shorted waveguides for geometries similar to the chamber foreseen for the upgrade of the Swiss Light Source (SLS 2.0). Initial tests utilized standard X-band waveguides at 12 GHz. To improve coating deposition, they used an ad hoc coating process for creating a dense or columnar type NEG coating (3–5 μm thick) on the waveguide walls. Even if the coating deposition can be well controlled for X-band waveguides, it presents limitations on the waveguide size in the case of characterizations of ultra-thin NEG layers requiring to move into the 100 GHz region. In [10,11], the EM characterization and impedance evaluation of standard NEG films was assessed in the sub-THz frequency domain by depositing roughly 3 μm of the material onto the lateral walls of a calibrated waveguide. Although this method can be extended to characterize other thin film coatings, it has its own disadvantages. These include issues such as local inhomogeneities like blistering and peeling, restrictions on sample size, and the inability to reuse the waveguide for subsequent measurements. Metallic waveguides in the THz region have been utilized as well to achieve high-resolution absorption spectra of molecular solids [12] and to analyze thin samples [13,14] using a technique known as time-domain waveguide spectroscopy [12,15]. In this approach, waveguides are designed to provide extended interaction lengths and highly confined electromagnetic fields [16], which significantly enhance sensitivity. Furthermore, the use of calibrated devices ensures the development of precise and reliable characterization methods. Although many of these experimental methodologies demonstrate excellent reliability and a high potentiality of THz waveguide spectroscopy with low losses, they are mainly focused on the optimization of the coupling of the input THz pulse with a single waveguide mode and of the guiding structure used for the characterization.

Moreover, the data indicate that coating parameters depend on the manufacturing process itself, affecting the surface impedance in various ways. As a consequence, we cannot assume that there is an ideal material surface to be characterized. There is the need for a technique that allows easily testing the technical surfaces representative of real samples. To overcome some of the problems related to metallic coatings, we developed a technique [17] to effectively evaluate the conductivity of metallic coatings in the sub-THz region by measuring pulsed signals passing through a specifically designed waveguide, where a central slab with the material under test is placed. The method is simple and reliable, and can be easily scaled in frequency allowing also the measurements of large area samples.

In this paper, we focused on testing technical surfaces that are representative of real samples. As previously mentioned, the impact of NEG coatings on beam dynamics depends on their conductivity, which is influenced by the manufacturing process [18]. In order to test the limits of our methodology, we characterized three different types of NEG coatings, with similar dimensions but different production methods, grown by sputtering on both sides of a copper slab at the CERN deposition facilities.

In Section 2.1, we present the methodology for the evaluation of the surface resistance of a multilayer vacuum chamber, based on the transmission line (TL) theory and previously validated on different layers [17,19]. The area of applicability of the model is also discussed.

In Section 2.2, we describe the experimental technique, based on THz time-domain spectroscopy, and give the details of the deposition procedure of three NEG coatings on a copper slab. For a reliable test of the NEG samples, the coating thickness required by the method is nearly an order of magnitude greater than what is commonly used in beam accelerator pipes.

In Section 3, the results of the EM characterization of the NEG samples in time and frequency domain are reported and discussed. The evaluated frequency range is suitable for synchrotron light sources [20] and for the Future Circular $e^{+}e^{-}$ Collider [6].

In the final section, we present a concise conclusion and discuss potential future applications of the time-domain waveguide technique.

## 2. Materials and Methods

### 2.1. The Methodology

**Figure 1:**
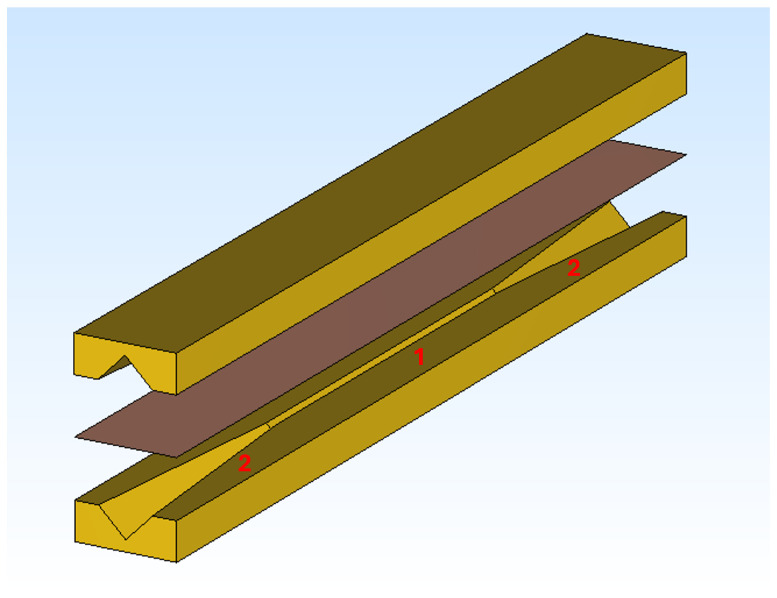
Three-dimensional rendering of a gold waveguide with diagonal guiding structure (1), two pyramidal horns (2), and a copper slab (the brownish plane) positioned at its center.

The material under test is grown on both sides of a copper slab, which is centrally placed in a specifically designed gold waveguide, as sketched in Figure 1. The central guiding structure has a diagonal (square rotated by $45^{\circ }$) shape. Two pyramidal horn antennas are used to collect and radiate a pulsed electromagnetic wave passing through the device. All measurements are taken in time domain and then transformed into frequency domain using a standard Fourier transform (FT) procedure.

To retrieve the conductivity value of the coated material, the amplitudes of the signal transmitted inside the device with a coated and with an uncoated slab (used as a reference) are compared. The guiding device can be decomposed in three parts: the central diagonal guiding structure and the two pyramidal horns. For each of these elements the values of the specific attenuation have been separately calculated as detailed in [19].

The final attenuation *A*, used for the calculations, is solely due to the presence of the slab in the waveguide, as the contribution from the attenuation of the uncoated walls is removed when estimating the relative attenuation (with respect to the reference signal). Specifically, the attenuation values, both in the diagonal waveguide $A_{\mathrm{diag}}$ and in the pyramidal transition $A_{\mathrm{pyr}}$, can be expressed by means of the following general formula considering the propagation along the slab as the sum of two modes ${\mathrm{TE}}_{1,0}$ and ${\mathrm{TE}}_{0,1}$ [21,22]:

(1) $$A=\frac{1}{2}Re\left(Z_{S}\right)\frac{\int {\left|n\times (H_{1,0}+H_{0,1})\right|}^{2}dl}{Re\left(Z_{1,0}\right){\left|I_{1,0}\right|}^{2}+Re\left(Z_{0,1}\right){\left|I_{0,1}\right|}^{2}},$$

where $Z_{i,j}$ is the i,j mode impedance and $I_{i,j}$ is the relevant excitation current. The field components are reported in [19] and are different for the two guiding elements, waveguide and transition. The surface impedance $Z_{S}$, resorting to the transmission line theory, can be expressed as follows:

(2) $$Z_{S}=Z_{\mathrm{coat}}\frac{Z_{\mathrm{cu}}+jZ_{\mathrm{coat}}\tan\left(k_{\mathrm{coat}}d\right)}{Z_{\mathrm{coat}}+jZ_{\mathrm{cu}}\tan\left(k_{\mathrm{coat}}d\right)},$$

where *d* is the coating thickness. $Z_{\mathrm{cu}}$ and $Z_{\mathrm{coat}}$ are the characteristic impedance of the copper and of the coating layer, respectively, evaluated in the Leontovich approximation for a metallic case ($\varepsilon^{\prime \prime }\gg \varepsilon^{\prime }$) [21]:

(3) $$Z=\left(1+j\right)\sqrt{\frac{\omega \mu }{2\sigma }}=\frac{1+j}{\sigma \delta },$$

$k_{\mathrm{coat}}$ is the propagation constant evaluated with the coating conductivity in the same metallic approximation:

(4) $$k=\left(1-j\right)\sqrt{\frac{\sigma \omega \mu }{2}}=\frac{1-j}{\delta },$$

δ is the skin-depth defined as follows:

(5) $$\delta =\sqrt{\frac{2}{\sigma \omega \mu }},$$

where μ is the total permeability, ω=2πf and σ the material conductivity. Adding the single-element contributions, we obtain the total relative attenuation:

(6) $$RA_{\mathrm{total}}=RA_{\mathrm{diag}}+2\;RA_{\mathrm{pyr}},$$

where $RA_{\mathrm{diag}}=A_{\mathrm{diag}}^{\mathrm{coat}}-A_{\mathrm{diag}}^{\mathrm{cu}}$ is the relative attenuation in the diagonal waveguide and $RA_{\mathrm{pyr}}=A_{\mathrm{pyr}}^{\mathrm{coat}}-A_{\mathrm{pyr}}^{\mathrm{cu}}$ is the relative attenuation in the single pyramidal transition.

Equation (6) gives the measure of the losses due to the coating material in the device through its overall length (horn antennas plus waveguide) and it is used for the evaluation of the conductivity of the material under test.

### 2.2. Electromagnetic Characterization

The setup is similar to the one used in [19], where the design of the guiding structure was optimized for the standard NEG coating, approximately 4 μm thick. The entire device under test (DUT), described in Section 2.1, is a parallelepiped of 16×12×140 mm^3^ (see Figure 1), containing the guiding structure, the two antennas, and the slab. DUT features and dimensions are reported in Table 1. For the pyramidal horns, maximum and minimum apertures along their length are indicated.

We took measurements on three different NEG coatings, plus the uncoated copper slab. The NEG growth process was performed at the CERN deposition facilities on both sides of the copper slabs, with three different methodologies. The first method used to realize the standard CERN NEG (TiZrV) sample was a DC magnetron sputtering technique at low pressure of Krypton [23]. The second methodology used to realize a densified coating was High Power Impulse Magnetron Sputtering (HiPIMS NEG sample), also performed at low pressure of Krypton. The third method for the deposition was DC magnetron sputtering conducted at high pressure of Krypton (High-Pressure NEG sample).

**Figure 2:**
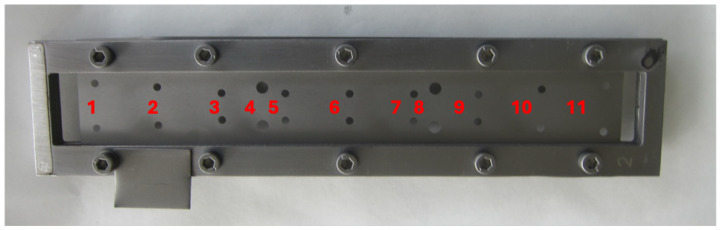
Top view of the coated slab surrounded by the aluminum frame used during the deposition. The holes are for the correct alignment of the slab in the waveguide during THz measurements. The red numbers are the position where the XRF thickness measurements are taken.

The dimensions of the slab are reported in Table 1. During the deposition process, in order to prevent the deformation, the slab is mounted in an aluminum frame (see Figure 2). The aluminum frame ensures that the slab is tightly held during the coating process and is removed just before the slab is placed inside the waveguide for measurements. The slab is secured in the waveguide using 18 + 4 positioning holes, 18 screws and 4 steady pins uniformly positioned along the waveguide to facilitate correct positioning and prevent possible deformation.

**Figure 3:**
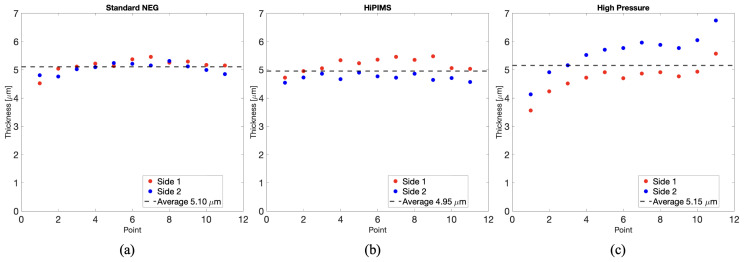
Measurements of the coating thickness on eleven points shown in Figure 2: (**a**) standard NEG, (**b**) HiPIMS NEG, (**c**) High-Pressure NEG.

Thickness measurements of the coating deposition were taken at the CERN deposition facilities, using X-Ray Fluorescence (XRF) at eleven different points along the median line of the slab (coinciding with the waveguide axis), shown in Figure 2. These measurements revealed a mean thickness of 5.10 μm for the standard NEG coated slab, 4.95 μm for the HiPIMS NEG coated slab, and 5.15 μm for the High-Pressure NEG coated sample (see Figure 3). For the first two samples, we can confirm the uniformity of the coating, as the maximum standard deviation is 0.24 μm. The third sample showed greater non-uniformity in the coating along the entire structure.

**Figure 4:**
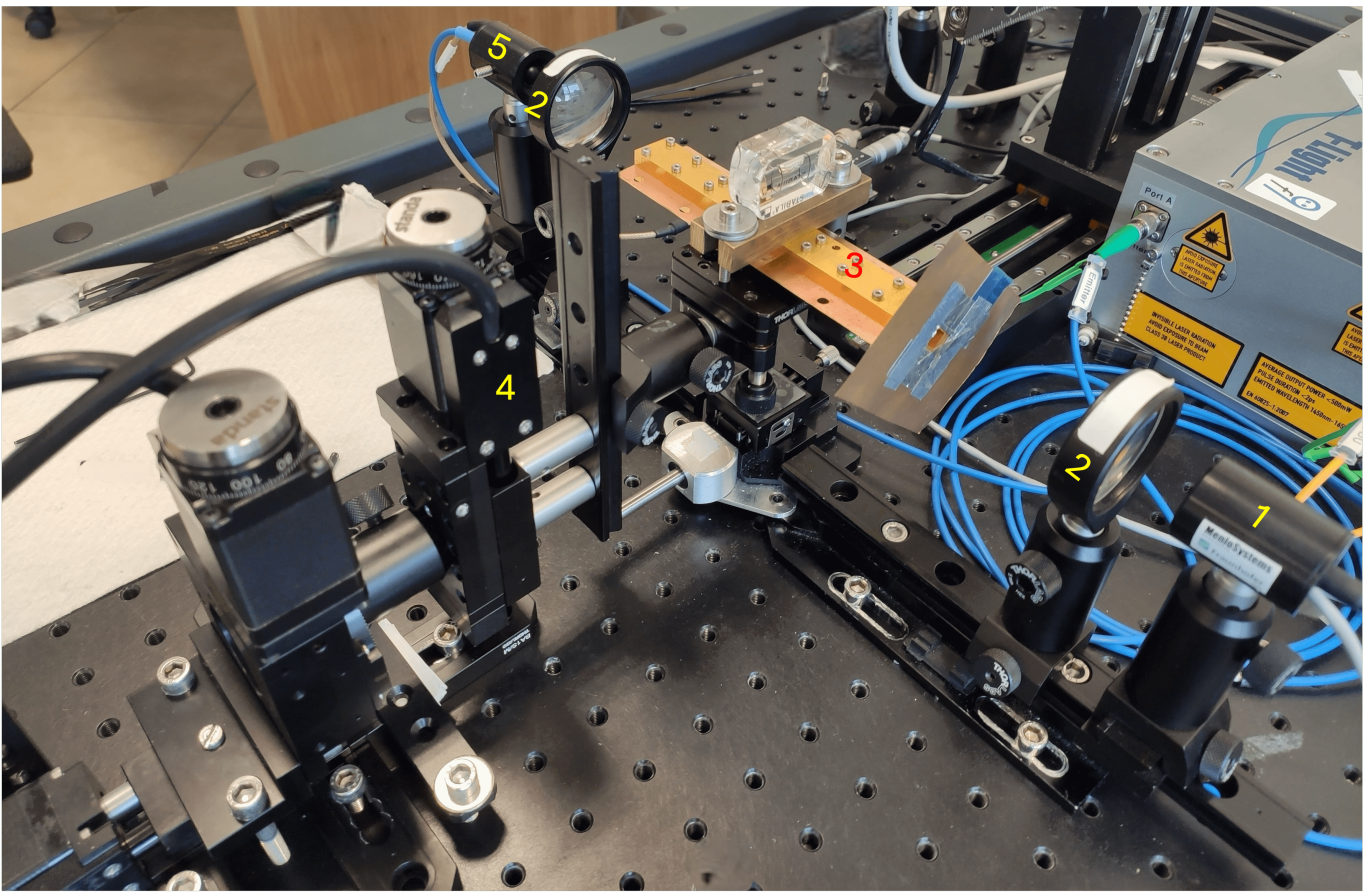
Photograph of the opto-mechanical setup: (1) emitter, (2) TPX collimating lens, (3) waveguide with embedded antennas, (4) micrometric alignment system, (5) detector.

To carry out sub-THz measurements, we employed a commercial Time Domain Spectrometer (TDS) operating in transmission mode (TERA K15 by Menlo Systems) and customized for the waveguide characterization [19] as shown in Figure 4. TPX (polymethylpentene) lenses were used to collimate the short (1–2 ps) linearly polarized pulse on the waveguide, producing a Gaussian-like beam with a waist of approximately 8 mm in diameter and a quasi-plane wave phase front.

**Figure 5:**
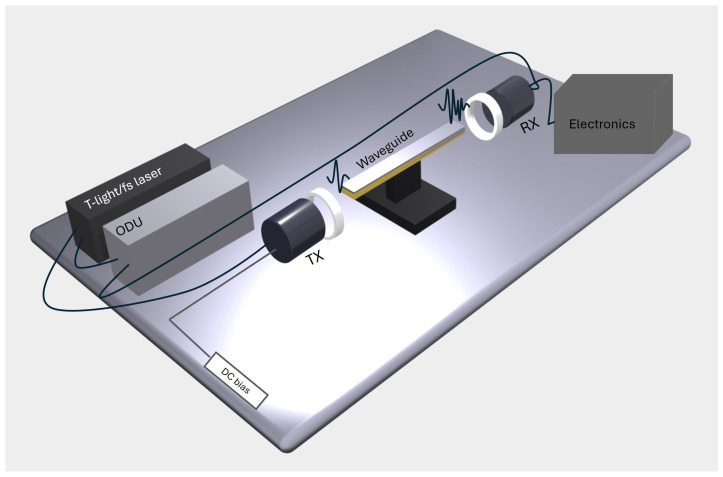
System framework diagram with key modules of the waveguide measurement system. ODU stands for optical delay unit, TX and RX are the emitter and the receiver, respectively.

Figure 5 shows the system framework diagram with key modules of the waveguide measurement system. In pump–probe mode, the laser output splits into two beams. Fiber-coupled photoconductive antennas are used for both the emission and detection of the electric field signal. An optical delay unit (ODU) is employed to control the time delay between the pump and probe beams.

The quasi-optical coupling between the free space signal and the input and output horn antennas is a critical aspect of the measurement. The use of a collimated THz beam ensures normal incidence on the waveguide entrance; meanwhile, a mechanical accurate alignment is performed fixing the lower part of the waveguide on a kinematic mount with a micrometric goniometer.

The THz beam is polarized with its electric field perpendicular to the waveguide slab so that the first excited modes are transverse electric (TE). The frequency window for a single mode (sum of the $TE_{1,0}$ and $TE_{0,1}$) propagation ranges from 135 GHz to 300 GHz, limited by the second propagating mode, the sum of $TE_{2,1}$ and $TE_{1,2}$ [24].

Coated slabs are inserted and replaced by removing only the top part of the parallelepiped. The electric field signal is recorded for each sample as a function of time by averaging 1000 pulses over a total acquisition time of 10 min to reduce the signal-to-noise ratio. Frequency-dependent transmission curves are derived using a standard FT algorithm. In the experiment, the frequency resolution is set to about 8 GHz, determined by the scanning range of the delay line.

## 3. Results and Discussion

The EM characterization in the sub-THz region of the different NEG coatings was performed resorting to the analytical tool described in Section 2.1. We preliminarily performed a parametric study of the relative attenuation as a function of frequency for different values of thickness and of coating conductivity in order to consider the variables that may affect the deposited layer quality. The results of these evaluations are detailed in [25].

**Figure 6:**
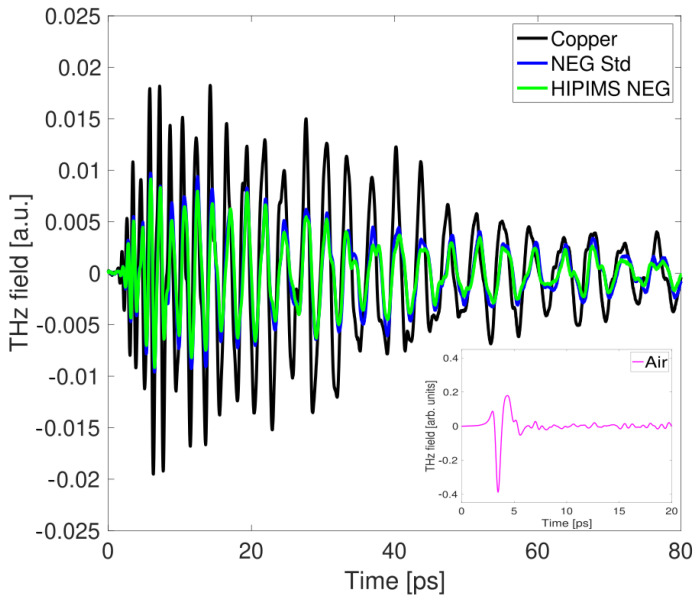
THz time- domain signal propagation in the device with copper slab only (black curve) and with the standard and HiPIMS NEG coated slabs (blue and green curves). In the inset, the THz signal propagating in free space is shown.

For each NEG sample, we first measured the bare copper slab, taken as reference. Figure 6 shows the comparison of the recorded THz signal transmitted through the guiding structure loaded with three different slabs: copper slab without coating, with standard NEG, and with HiPIMS NEG coating, respectively. Because of the broadband nature of the THz pulse and the presence of the low-frequency cutoff for the $TE_{1}$ mode, the frequency components suffer a high group velocity dispersion near the cutoff, resulting in a severe distortion of the electric field waveform. When passing through the 62 mm long metallic waveguide, the 1–2 ps free space signal (see inset of Figure 6) is stretched to more than 80 ps with about 30 major cycles of oscillation. For all the three samples, the time-domain signal exhibits the same negative chirp, since high frequencies travel faster in the waveguide, arriving earlier in time, compared to low frequencies [15]. In both cases with coated slabs, the signal attenuation with respect to the bare copper slab can be clearly observed throughout the entire time interval considered for the evaluation.

**Figure 7:**
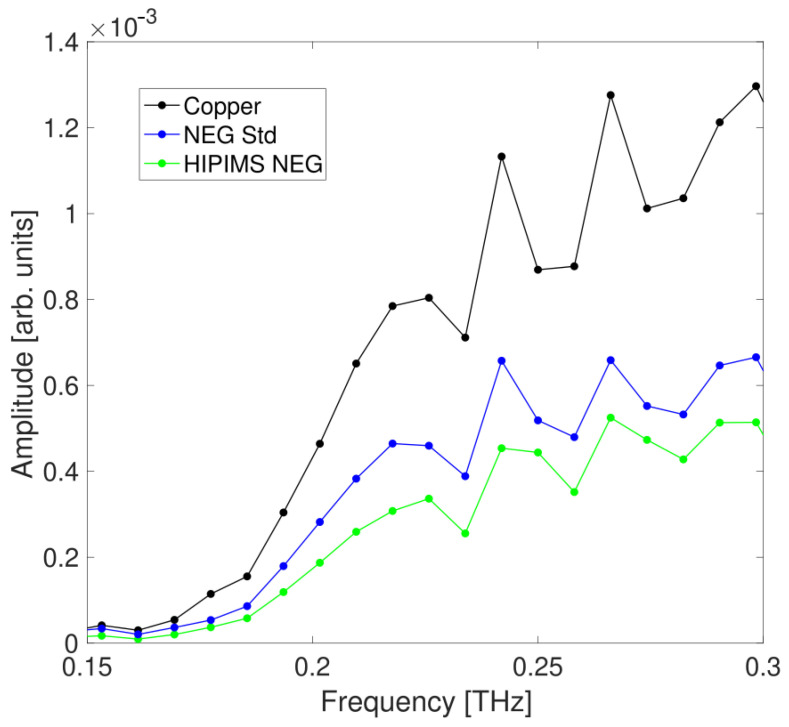
Frequency spectrum showing the averaged amplitude data for the waveguide with the copper slab (black dots) and with the standard and HiPIMS NEG coated slabs (blue and green dots).

The amplitude spectra vs. frequency after FT are presented in Figure 7 for the two NEG samples compared with the bare copper bulk.

The data in Figure 7 represent the average value of five different measurements, each time repositioning the upper part of the waveguide and the slab. This procedure is performed to reduce the unavoidable measurement uncertainty, which is estimated from the variance of repetitive measurements. The data are shown up to the frequency where single-mode propagation in the waveguide is maintained, ensuring there is no interference from higher-order modes that can alter the field distribution. Above 300 GHz, a second mode, given by the sum of $TE_{2,1}$ and $TE_{1,2}$ [24], starts propagating and prevents the monomodal analysis. For the retrieval of the surface impedance, data below 190 GHz have been discarded to avoid artifacts in the spectrum caused by group and phase velocity dispersion, which are especially pronounced near the cutoff frequency, visible at approximately 150 GHz in the figure. In the graph, the trend is similar for all three curves with a marked difference in amplitude for each individual sample.

**Figure 8:**
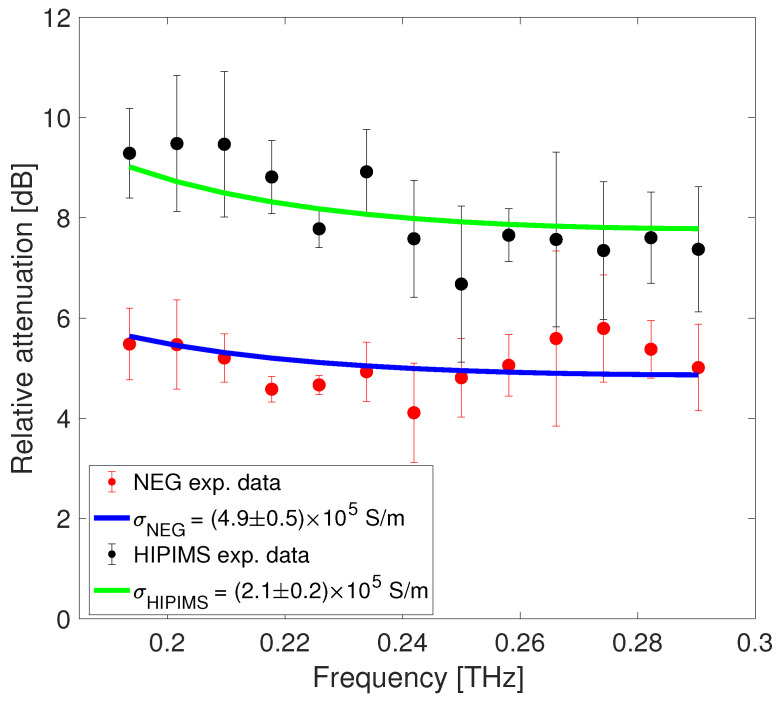
Experimental relative attenuation as a function of frequency on the standard and HiPIMS NEG coated slab (red and black dots, respectively) and best-fit curves (blue and green lines respectively).

The third step of our analysis is the evaluation of the relative attenuation, caused by the coating material, performed by comparing the coated slab frequency spectra with the uncoated reference slab one. This evaluation considers the losses along the slab produced throughout the device entire length, including the horn antennas and waveguide. Figure 8 shows the measured relative attenuation for both the standard and HiPIMS NEG coatings (represented by red and black dots, respectively) compared to the copper reference within the frequency range of 190 to 290 GHz. Counter-intuitively, attenuation decreases for all frequencies above the cutoff, which is a peculiar feature of the $TE_{1}$ mode propagation in the metallic rectangular waveguide [26].

The experimental data were interpolated using a best-fit curve (see Equation (6)), employing a nonlinear regression method based on the least squares approach, with the NEG conductivity treated as the unknown variable. The fitting procedure resulted in $\sigma_{\mathrm{NEG}}=\left(4.9\pm 0.5\right)\times 10^{5}$ S/m for the standard NEG and $\sigma_{\mathrm{HiPIMS}}=\left(2.1\pm 0.2\right)\times 10^{5}$ S/m for the HiPIMS NEG sample. The green and blue lines represent the analytically evaluated relative attenuation for the two estimated conductivities. The observed difference between the two samples might indicate increased disorder, leading to poorer transport properties and, therefore, lower conductivity. Referring to [27], the effect of sample roughness on the estimated NEG conductivity was evaluated. Assuming an average roughness of 0.2 μm for our samples [10,28], this gives a maximum conductivity reduction that lies within the measurement error band for our frequency range.

**Figure 9:**
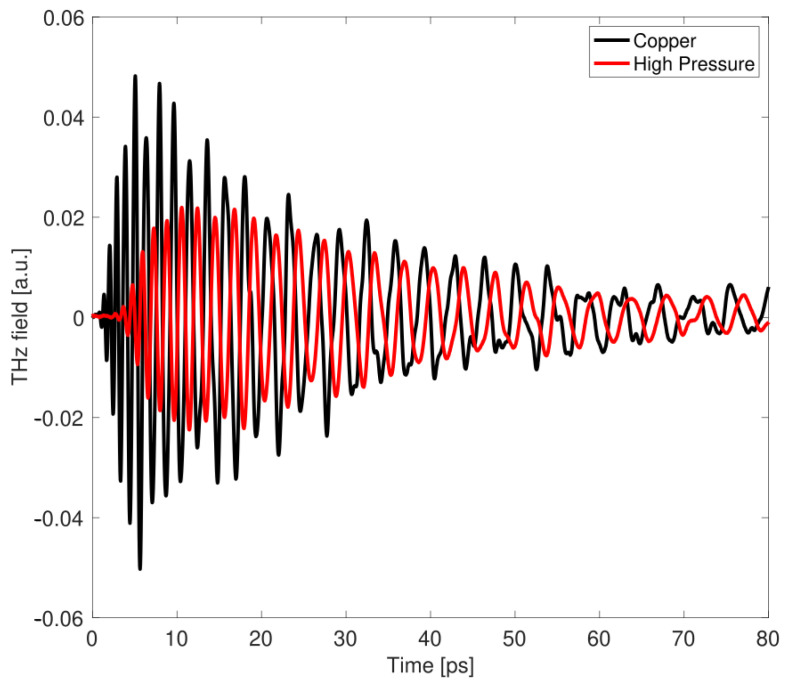
THz time- domain signal propagation in the device with copper slab only (black curve) and with the High-Pressure NEG coated slab (red curve).

Concerning the estimation of conductivity for the third sample (High-Pressure NEG), unfortunately, our current method fails to deliver a dependable value. In Figure 9, one can observe for this coating not only a signal attenuation, but also a temporal shift of roughly 2.1 ps with respect to copper and an oscillation period slightly different from that of the bare slab case, which is not observed in the other NEG samples. This shift might be an indication that there is a variation in the complex coupling coefficient of the electric field passing through the two different slabs [29] and a difference between the propagation (phase) velocity through the copper and the High-Pressure NEG coated waveguide. Assuming single-mode propagation, a change in the group velocity distortion modifies the down-chirp effect on the transmitted signal waveform [15].

Comparing the time-domain response of Figure 6 and Figure 9, one can see that the time response of the High-Pressure NEG sample is significantly different from what is observed in both the standard and HiPIMS NEG coatings. The signal is strongly attenuated in the initial part of the chirped pulse (higher frequencies), progressively approaching the behavior shown by the bare copper slab at longer times (lower frequencies). This implies that, after Fourier transforming, in the specific frequency range of monomodal propagation analysis (190–290 GHz), the attenuation in the High-Pressure NEG is comparable to the reference sample one. Therefore, Equation (6) cannot be effectively applied and as a consequence a reliable conductivity value retrieved. A possible explanation is that the material conductivity is $\sigma_{\mathrm{High}\;\mathrm{Pressure}}\leq 10^{4}$ S/m, and the signal at those frequencies predominantly reflects the presence of copper rather than the coating material.

In Table 2, we summarize the conductivity of different NEG samples measured with various methodologies. The wide range of experimental values, spanning almost two orders of magnitude, underlines how the NEG properties depend on sample manufacturing and deposition conditions, and the importance of testing technical surfaces which are representative of samples grown under real operational conditions.

Our results for the standard NEG (TiZrV) are in good agreement with previous data obtained on different standard NEG samples [17,19] and with values extracted in the frequency domain [10]. Moreover, the results shows that our methodology is capable of discriminating between two different NEG samples (standard and HiPIMS), with similar thickness, but slightly different conductivity.

## 4. Conclusions

In this paper, we employed a time-domain method based on a transmission line model and a sub-THz waveguide to characterize the electromagnetic properties of technical coated surfaces that are representative of real walls used in the vacuum pipes of particle accelerators. To test the limits of our methodology, we characterized three different types of NEG coatings, all with similar dimensions and deposited on both sides of a copper slab under different sputtering conditions at the CERN deposition facilities: standard NEG, HiPIMS NEG, and High-Pressure NEG.

To assess the conductivity of the coatings, the technique was applied in three steps: acquiring the transmitted time-domain signals of the coated samples and of the reference (a copper slab without coating); analyzing the corresponding frequency-domain response obtained through Fourier transformation; and evaluating the experimental relative attenuation compared to the reference sample. The data were interpolated with a best-fit curve, treating the NEG conductivity as the unknown variable, which allowed us to evaluate the conductivity of the standard NEG and HiPIMS coatings.

For the tested samples, we obtained $\sigma_{\mathrm{NEG}}=\left(4.9\pm 0.5\right)\times 10^{5}$ S/m for the standard NEG and $\sigma_{\mathrm{HiPIMS}}=\left(2.1\pm 0.2\right)\times 10^{5}$ S/m for the HiPIMS NEG sample. These results show that we are able to discriminate between two different NEG samples with similar thickness but slightly different conductivity. The experimental data are in good agreement with other measurements taken on different standard NEG samples and with values obtained in the frequency domain on similar standard NEG coatings.

Having accurate values of $\sigma_{coat}$ under operating conditions (coating deposited on a metallic slab) is extremely useful for evaluating the real part of the surface impedance as a function of frequency, resorting to Equation (2). This information is currently used for modeling the resistive wall component of the beam impedance in modern accelerators.

For the High-Pressure NEG sample, we observed in the time signal a shift of approximately 2.1 ps with respect to the signal transmitted through the waveguide with the copper slab (the reference signal), as well as a difference in the down-chirp effect, which was not observed in the other coated samples. We believe this is due to the poorer response of the High-Pressure NEG coating, likely resulting from lower conductivity ($\sigma_{\mathrm{High}\;\mathrm{Pressure}}\leq 10^{4}$ S/m), which affects the way the signal propagates through the waveguide, altering both the phase and group velocity at the frequencies of interest. In this case, we could not reliably apply our analytical model within our specified frequency range (190–290 GHz).

Compared to other waveguide methodologies, our approach selectively excites only two chosen degenerate waveguide modes, operating within a known modal configuration that can be analytically described. The methodology allows the use of different coating samples without changing the waveguide, and alternative geometries and dimensions of the waveguide can be also considered, so that one can work in different frequency ranges. However, it is important to note that reducing the size of the guiding structure makes coupling with the passing-through signal more challenging. Our methodology proves to be a reliable tool for easily conducting EM testing on technical surfaces that are representative of real samples. The technique seems to have an inherent limitation when the response of the waveguide with the coating material is lower than that of the reference device, requiring the extension of the frequency range of analysis to include higher-order modes and accurately assessing their contributions.

## Figures and Tables

**Table 1:** Technical specifications of device under test and sample.

Waveguide material	Brass (Au plated)
Guiding structure	Diagonal
Length [mm]	62
Side [mm]	1.1
Transition	Pyramidal Horns
Length [mm]	39
Side (min and max apertures [mm]	1.1→6
Total length [mm]	140
Sample	Slab
Material	Copper (+NEG)
Length [mm]	140
Thickness [μm]	50 (+coating)

**Table 2:** Conductivity measurements of various NEG coatings.

Coating	Thickness [μm]	σ [S/m]	Method and Frequency	Reference
Columnar NEG	0.93–24.5	$1.4\times 10^{4}$	RF cavity (7.8 GHz)	[8]
Dense NEG	0.76–14.5	$8\times 10^{5}$	RF cavity (7.8 GHz)	[8]
TiZrV NEG	1.38	$9.35\times 10^{4}$	Four-probe technique (DC)	[30]
TiZrV-Cu NEG	1.62	$3.24\times 10^{5}$	Four-probe technique (DC)	[30]
TiZrV NEG	3 non-homogeneous	$2.4\times 10^{5}$	WG in FD (500–750 GHz)	[10]
TiZrV NEG	3 non-homogeneous	$3.5\times 10^{5}$	WG in FD (220–340 GHz)	[10]
TiZrV NEG	9	$1\times 10^{6}$	WG in FD (10 GHz)	[10]
TiZrV NEG	3.8	$7.7\times 10^{5}$	WG in TD (190–300 GHz)	[19]
TiZrV NEG	4.3	$4.2\times 10^{5}$	WG in TD (190–300 GHz)	[19]
TiZrV NEG	5.1	$4.9\times 10^{5}$	WG in TD (190–300 GHz)	This work
HiPIMS NEG	4.95	$2\times 10^{5}$	WG in TD (190–300 GHz)	This work

## Data Availability

Data are contained within the article.
